# Roles of tumor-associated macrophages in anti-PD-1/PD-L1 immunotherapy for solid cancers

**DOI:** 10.1186/s12943-023-01725-x

**Published:** 2023-03-21

**Authors:** Hao Zhang, Lin Liu, Jinbo Liu, Pengyuan Dang, Shengyun Hu, Weitang Yuan, Zhenqiang Sun, Yang Liu, Chengzeng Wang

**Affiliations:** 1 Department of Colorectal Surgery, The First Affiliated Hospital of Zhengzhou University, Zhengzhou, 450001 China; 2 Henan Institute of Interconnected Intelligent Health Management, The First Affiliated Hospital of Zhengzhou University, Zhengzhou, 450052 Henan China; 3 Department of Ultrasound, The First Affiliated Hospital of Zhengzhou University, Zhengzhou, 450052 Henan China; 4 Department of Radiotherapy, Henan Cancer Hospital, Affiliated Cancer Hospital of Zhengzhou University, Zhengzhou, 450001 China

**Keywords:** Tumor-associated macrophages, Immune checkpoint inhibitors, Cancer, Combined therapy

## Abstract

In recent years, tumor immunotherapy has made significant progress. However, tumor immunotherapy, particularly immune checkpoint inhibitors (e.g., PD-1/PD-L1 inhibitors), benefits only a tiny proportion of patients in solid cancers. The tumor microenvironment (TME) acts a significant role in tumor immunotherapy. Studies reported that tumor-associated macrophages (TAMs), as one of the main components of TME, seriously affected the therapeutic effect of PD-1/PD-L1 inhibitors. In this review, we analyzed TAMs from epigenetic and single-cell perspectives and introduced the role and mechanisms of TAMs in anti-programmed death protein 1(anti-PD-1) therapy. In addition, we summarized combination regimens that enhance the efficacy of tumor PD-1/PD-L1 inhibitors and elaborated on the role of the TAMs in different solid cancers. Eventually, the clinical value of TAMs by influencing the therapeutic effect of tumor PD-1/PD-L1 inhibitors was discussed. These above are beneficial to elucidate poor therapeutic effect of PD-1/PD-L1 inhibitors in solid tumors from the point of view of TAMs and explore the strategies to improve its objective remission rate of solid cancers.

## Background

Since the US Food and Drug Administration approved ipilimumab (anti-CTLA-4) for the treatment of metastatic melanoma in 2011 [1], several checkpoint-blocking therapies targeting the PD-1/PD-L1 axis have been approved for the treatment of multiple tumor types. A good understanding of the impact of the tumor microenvironment(TME) on tumor immunotherapy is essential for effectively integrating immunotherapy with chemotherapy, targeted therapy, and other immunotherapies. Studies have shown that tumor-associated macrophages (TAMs) act a crucial role in tumor immunotherapy [2]. TAMs affect the therapeutic effect of PD-1/PD-L1 inhibitors through various mechanisms, including regulating the expression of PD-L1 in tumor cells and secreting a variety of cytokines to produce tumor-promoting TME [3–5]. Chemotherapy, targeted therapy, and radiotherapy effectively remodel TME, especially TAMs, and transform them from pro-tumor to antitumor [6–8].

In this review, we summarized the currently approved immune checkpoint inhibitors (ICIs) (Table 1) and the role of TAMs in anti-PD-1/PD-L1 treatment for solid cancers [9–16]. We described the synergistic effects of anti-PD-1/PD-L1 therapy in combination with targeted therapy, chemotherapy, radiotherapy, and other immunotherapies. The mechanism of the impact of TAMs on immunotherapy in different solid cancers was also concluded. Furthermore, the clinical application value of TAMs for the solid cancer treatment of PD-1/PD-L1 inhibitors is proposed based on the vital role of TAMs in immunotherapy.

**Table 1:** The approved immune checkpoint inhibitors in the globe

Target	Active Ingredients	First approval time	Company	Application(approved in the globe)
PD-1	Nivolumab	2014	Bristol Myers Squibb	Melanoma, NSCLC, MPM, RCC, HL HNSCC, UC, CRC, HCC, Esophageal Cancer, GC, GEJC, EA
	Pembrolizumab	2014	Merck Sharp Dohme	Melanoma, NSCLC, HNSCC, HL, PMBCL, UC, CRC, GC, Esophageal cancer, CC, HCC, MCC, RCC, Endometrial carcinoma, CSCC, TNBC
	Cemiplimab	2018	Regeneron Pharmaceuticals	CSCC, BCC, NSCLC
	Toripalimab	2018	Shanghai Junshi Biosciences Co., Ltd.	Melanoma, UC, NPC, ESCC
	Sintilimab	2018	Innovent Biologics, Inc	NSCLC, HL, HCC, ESCC, GC, GEJC
	Camrelizumab	2019	Jiangsu Hengrui Medicine Co.,Ltd.	NSCLC, HL, HCC, ESCC, NPC
	Tislelizumab	2019	Beigene, Ltd.	NSCLC, HL, UC, HCC, NPC, ESCC, CRC
	Zimberelimab	2021	Guangzhou Gloria Biosciences Co., Ltd.	HL
	Prolgolimab	2020	Biocad.	Melanoma, SC
	Dostarlimab	2021	GSK Plc	Endometrial carcinoma
PD-L1	Atezolizumab	2016	Genetech Inc	Melanoma, NSCLC, SCLC, HCC, ASPS
	Durvalumab	2017	AstraZeneca	NSCLC, ES-SCLC, BTC, HCC
	Avelumab	2017	EMD Serono Inc	UC, MCC, RCC
CTLA-4	Ipilimumab	2011	Bristol Myers Squibb	Melanoma, RCC, CRC, HCC, NSCLC, MPM, Esophageal cancer

*Abbreviations*: *ASPS* Alveolar soft part sarcoma, *BCC* Basal cell carcinoma, *BTC* Biliary tract cancer, *CC* Cervical cancer, *CRC* Colorectal cancer, *CSCC* Cutaneous squamous cell carcinoma, *EA* Esophageal adenocarcinoma, *ESCC* Esophageal squamous cell carcinoma, *ES-SCLC* Extensive-stage small cell lung cancer, *GC* Gastric cancer, *GEJC* Gastroesophageal junction cancer, *HCC* Hepatocellular carcinoma, *HL* Hodgkin lymphoma, *HNSCC* Head and neck squamous cell cancer, *MCC* Merkel cell carcinoma, *MPM* Malignant pleural mesothelioma, *NPC* Nasopharyngeal carcinoma, *NSCLC* Non-small cell lung cancer, *PMBL* Primary mediastinal B cell lymphoma, *RCC* Renal cell carcinoma, *SC* Skin cancer, *SCLC* Small cell lung cancer, *TNBC* Triple-negative breast cancer, *UC* Urothelial carcinoma

## TAMs and TME

TME contains not only tumor cells but also innate and adaptive immune cells, fibroblasts, endothelial cells, pericytes, and non-cellular components such as extracellular matrix and soluble signals that infiltrate the tumor [17, 18]. TME is reportedly deeply associated with tumor tissue formation, survival, and metastasis [19, 20]. TAMs are the most plasticity and the highest proportion of immune cells in the TME [21]. TAMs were generally classified into two main phenotypes: classical activation (M1-like macrophages) and alternating activation (M2-like macrophages) [22]. M1-like macrophages are low in mannose receptor (CD206) and high expression of MHCII. M1-like macrophages are characterized by increased expression of inducible nitric oxide synthase (iNOS), tumor necrosis factor-α(TNF-α), and co-stimulatory molecules such as CD40, CD86, and various pro-inflammatory cytokines such as IL6, IL1b, IL12a, IL12b. M1-like macrophages induce antitumor immune responses through their T cell stimulating activity [23, 24]. TAMs were polarized to the M2 type under the induction of a variety of mediators, including IL-4, IL-10, transforming growth factor-β (TGF-β), and macrophage colony-stimulating factor (M-CSF) [24, 25]. Unlike M1, CD163^+^ is characteristic of M2-like macrophages, which express high mannose receptors and low levels of MHC II and release immunosuppressive cytokines such as vascular endothelial growth factor (VEGF) and arginase 1 (Arg-1), IL-10, TGF-β, indolamine 2,3-dioxygenase (IDO). In terms of cell function, M2-like macrophages promote tumor immune evasion, angiogenesis, tumor growth and metastasis [26–32].

Increasing evidences suggest that TAMs play a significant role in tumor development. TAMs directly communicate with tumor cells. On the one hand, TAMs affect tumor cells through exosome metastasis of substances like some non-coding RNAs(ncRNAs) [33]. TAMs-derived miR-223 is vital for breast cancer progression. Similarly, both miR-21-5p and miR-155-5p act essential roles in the migration and invasion processes of colon cancer cells [34]. In addition, lncRNA SBF2-AS1 absorbed by pancreatic cancer cells also promotes tumor proliferation, invasion, and migration [35]. Parallelly, the TAMs-derived VEGF and miR-501-3p directly mediate the angiogenesis in the tumor tissues [36, 37]. Furthermore, the miR-365 exosomes inhibit the effects of gemcitabine by upregulating pyrimidine metabolism and increasing nucleotide triphosphate levels in cancer cells [38]. On the other hand, TAMs secrete various cytokines that act on tumor cells. For example, TNF, IL-6, and IFN- γ upregulate PD-L1 expression in tumor cells. Indirect effects of TAMs on tumor cells are achieved by influencing other immune cells to regulate the TME. TAMs directly inhibit CD8^+^ T-cell proliferation through the metabolism of L-arginine via Arg-1, iNOS, and oxygen radicals [39, 40]. TAMs also induce T cell inhibition by the immune checkpoint through upregulation of PD-L1 expression. Moreover, TAMs recruit Tregs through CCL22 to further inhibit the antitumor immune response of T cells [41]. M2-polarized TAMs release a variety of anti-inflammatory cytokines (e. g., TGFB1) and chemokines (e. g., CCL22) that inhibit dendritic cell maturation and thus limit antigen presentation [41].

**Fig. 1:**
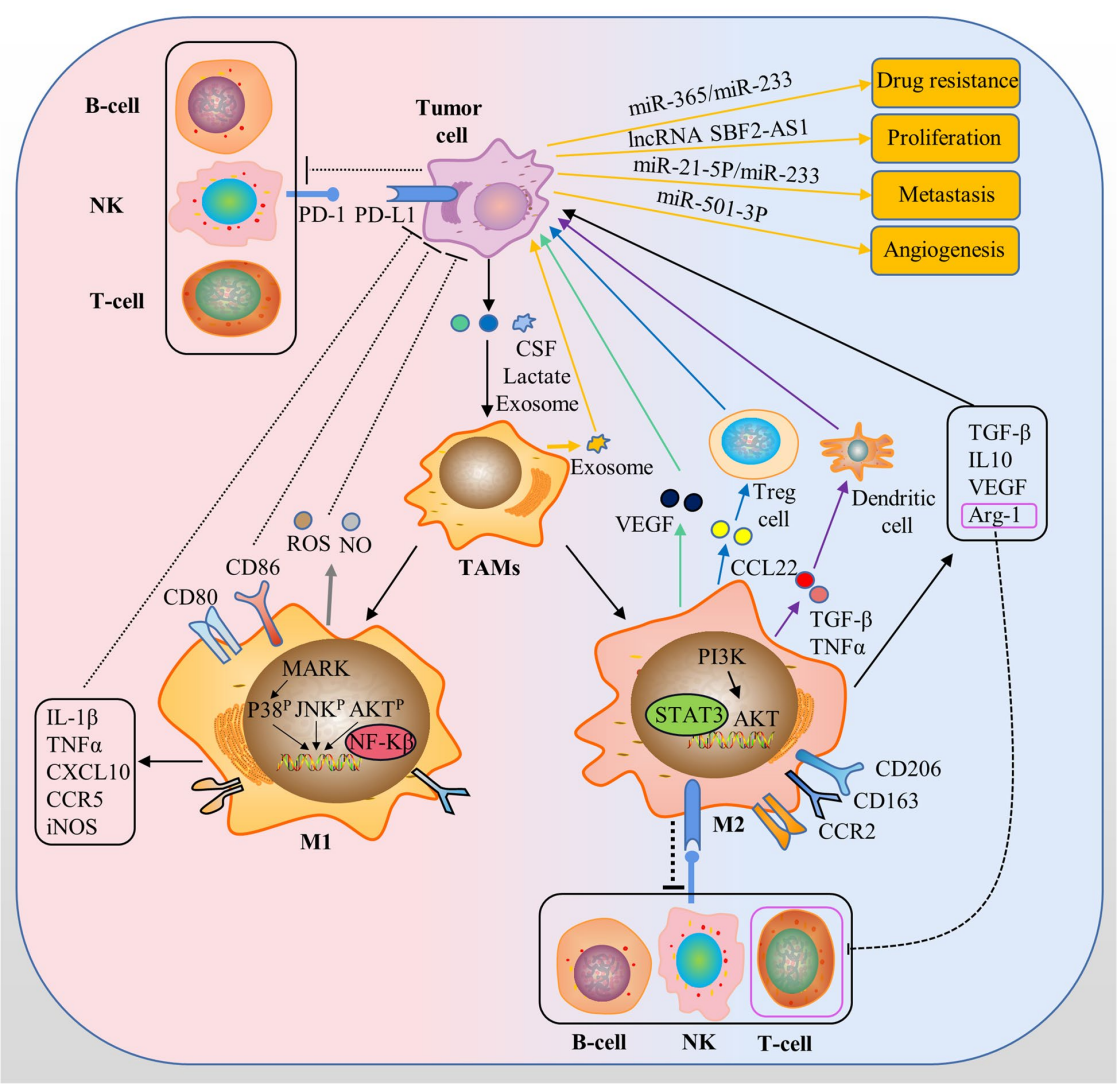
The role of TAMs in TME. Exosomes derived from TAMs deliver various molecules into tumor cells, which contributes to tumor development. Exosomal lncRNA SBF2-AS1 facilitates tumor cell proliferation. Exosomal miR-223 and miR-21-5p promote the metastasis of tumor cells from the primary tumor to the distal organs. Exosomal miR-501-3p promotes the angiogenesis of tumors. Exosomal miR-223 and miR-365 help tumor cells develop resistance to chemotherapy. TAMs express ligand receptors for PD-1 and CTLA-4, inhibiting the cytotoxic function of T cells, NK cells, and NK cells upon activation. TAMs express chemokine CCL22, etc., to recruit Treg cells. TAMs secrete VEGF to promote angiogenesis in TME. TAMs release a variety of anti-inflammatory cytokines to inhibit dendritic cell maturation, thereby limiting antigen presentation. In addition, tumor cells affect TAMs polarization by releasing exosomes, cytokines and their metabolites

Evidently, TAMs create a TME suitable for tumor growth by suppressing the antitumor activity of immune cells. Inversely, when TAMs are polarized to M1, they directly mediate cytotoxicity to kill tumor cells. In this case, macrophages release tumor-killer molecules, such as reactive oxygen species (ROS) and NO, which have cytotoxic effects on tumor cells [42]. The other is antibody-dependent cell-mediated cytotoxicity killing of tumor cells that requires the involvement of antitumor antibodies [43]. At the same time, the effect of TAMs on cancer cells is not unidirectional. Tumor cells also regulate TAMs to exert an immunosuppressive function through multiple mechanisms. For example, colony-stimulating factor 1 (CSF1) secreted by tumor cells favors the recruitment of monocyte-derived macrophages to the TME and polarizes them to the M2-like manner [44]. Moreover, lactate produced due to the high metabolism of tumor cells promotes M2 polarization of TAMs [45]. Consequently, TAMs build a complex immune regulatory network through various signaling mechanisms and other cells in the TME (Fig. 1).

## ICIs of cancer therapy

In the 90s of the twentieth century, immune checkpoint molecules were discovered, and two representative checkpoint pathways were cytotoxic T lymphocyte-associated protein 4 (CTLA-4) and PD-1 [46, 47]. Under physiological conditions, immune checkpoints inhibit the overactivation of T cells and prevent autoimmune responses, which is an essential mechanism for maintaining the immune balance in the body. Among them, CTLA-4 is induced to be expressed on activated T cells, competing with CD28 to inhibit CD80/CD86-mediated synergistic stimulation signals. In addition, antigen-stimulated T cells upregulate the expression of PD-1 to bind to their ligand PD-L1 or programmed death ligand 2 (PD-L2), inhibiting T cell overactivation [48–50]. However, the mechanism of maintaining immune balance has become an accomplice to the tumor in the tumor state. The reason of the above is that some cancer cells strongly express ligands of immune checkpoint molecules, such as PD-L1 and PD-L2. These ligands bind to PD-1 in activated T cells, specific B cells, natural killer cells (NKs), dendritic cells (DCs), and macrophages. The interaction between PD-1 and PD-L1 significantly inhibits the antitumor immunity of cytotoxic T cells, producing immunosuppressive effects and thus causing the immune escape phenomenon of tumors [51–53]. By contrast, PD-1 is expressed more widely in various immune cells than CTLA-4, which is limited to T cells, meaning that PD-1 may play a broader role in immunomodulation.

ICIs eliminate the suppressive signals of T cell activation, consequently enabling tumor-reactive T cells to overcome regulatory mechanisms and producing a potent antitumor response [54]. To date, all approved ICIs are monoclonal antibodies (Table 1) that block CTLA-4, PD-1, or PD-L1, essential drugs for activating T cells to promote their immune function [54]. However, the efficacy of ICIs is often limited and transient, reflecting the complexity of antitumor immunity [55, 56]. For example, ICIs are less effective in treating cancers of microsatellite stability of wild gene abnormalities which don’t induce cancer antigen-specific T cells. Furthermore, immunosuppressive TME also affects the clinical effect of ICIs. Meanwhile, the effectiveness of immunotherapy is affected by various resistance mechanisms, including immune rejection, immunoediting, antigen presentation reduction, and immunosuppression of soluble cellular [57, 58]. Notably, TAMs are key to these mechanisms. Because TAMs are an important inducing factor for inhibitory TME and a fundamental reason for the resistance to ICIs. Specifically, TAMs capture anti-PD-1/PD-L1 through Fcg receptors present on the cell surface [59]. In addition, TAMs upregulate PD-L1 expression in tumor cells and other immunosuppressive cells by secreting cytokines and metabolites [60, 61]. Furthermore, TAMs directly express PD-L1 under the influence of TME state, and induce CD8^+^ T cell inactivation and apoptosis through PD-1 binding on the surface of CD8^+^ T cells [62]. In this case, treatment with anti-PD-1/PD-L1 will be less effective. In this state, if TAMs can be prevented from expressing PD-L1, it will be beneficial to the therapeutic effect of ICIs.

## Analysis of TAMs from epigenetic perspectives

Epigenetics refers to heritable changes in gene function without altering the DNA sequence of a gene. Its mechanisms of action include, but are not limited to, DNA methylation, histone modification and ncRNAs [63]. Studies have shown that the epigenetic regulation of TAMs is essential for their differentiation and functional programming [64, 65].

### Effect of DNA methylation

DNA methylation refers to silencing gene transcription and is characterized by the transfer of methyl groups to the cytosine ring of DNA (forming 5-methylcytosine) by DNA methyltransferases (DNMTs) [66]. DNA methylation is removed by another set of enzymes known as ten-eleven translocation proteins [67]. Studies have shown that DNMT3b knockdown induces M2 polarization and increases the expression of M2-like macrophages markers, such as Arg-1 and mannose receptor type C. Moreover, overexpression of DNMT3b inhibits the expression of IL-4-induced Arg-1 in macrophages. This suggests that DNMT3b is a vital factor in inhibiting macrophage polarization to M2 [68].

### Effect of histone modification

Common histone modifications include histone acetylation-regulated histone acetylation and histone methyltransferase-mediated histone methylation [69].

Histone acetylation promotes gene transcription, which is mediated by histone acetyltransferase (HATs) and removed by histone deacetylase (HDACs). Both of them play an integral role in gene expression regulation. For example, autocrine IFN-β–Jak–STAT loops induced by Toll-like receptor (TLR) ligands and TNF are crucial links in M1 activation. The response of molecules downstream of IFN is strongly dependent on HDAC3 [25, 70]. A similar situation occurs in the mechanism of action of CCCTC-binding factor (CTCF). CTCF is a crucial transcription factor in TAMs. CTCF forms a complex with PACERR (an antisense LncRNA) to recruit HAT to the promoter region of its downstream gene PTGS2 (a tumor-promoting M2 gene). HAT induces histone acetylation and chromatin accessibility, promoting their expression and ultimately affecting M2 differentiation. Meanwhile, HAT enhances the pro-tumor metastasis effect of M2-like macrophages [71]. Similarly, HDAC is a negative regulator of M2 polarization, and HDAC9 deletion leads to the downregulation of inflammatory genes and M2 polarization [72]. Furthermore, HDAC6 intervention reduces the anti-inflammatory phenotype of TAMs. M2 polarization was inhibited after the inhibition of HDAC6 enzyme activity with the drug and increased M1 polarization [73].

Histone methylation is facilitated by histone methyl transferases and removed by histone demethylase. H3K27 methylation is a mark for transcription repression. After IL-4 treatment, H3K27 me2/3 was significantly reduced at the promoter of the M2 marker gene (i.e., Arg-1). Meanwhile, H3K27me2/3-specific demethylase Jmjd3 was significantly elevated under IL-4 induction. Jmjd3 helps keep the M2 marker gene in active state [74].

### Effect of ncRNAs

NcRNAs play a significant role in the post-transcriptional control of gene expression [75]. The epigenetic remodeling by ncRNAs regulates macrophage activation and functional programming. Among them, ncRNAs that play a key role are mainly divided into three categories: microRNA (miRNA), circular RNA (circRNA), and long noncoding RNA (lncRNA). Firstly, miRNAs are small regulatory RNA molecules that modulate the expression of their target genes [76]. MiRNAs play a huge regulatory role in the gene expression and polarization processes of macrophages. Some of them induce an antitumor immune microenvironment. For example, miR-98 regulates macrophage polarization from M2-like macrophages to M1-like macrophages in hepatocellular cancer (HCC) by targeting IL-10 and induces elevated expression levels of M1-like macrophages marker cytokines, such as TNF-α, IL-1β, and IL-12 [77]. Similarly, miR-101 directly targets DUSP1 to regulate MAPKs activation and subsequent pro-inflammatory cytokines production [78]. In addition, miR-17a and miR-20a also induce M1 polarization and activate M1-like macrophages [79]. In addition, some miRNAs inhibit M2 polarization through various pathways, such as miR-155, miR-720, MiR-23a, and miR-127etc [80–83]. Moreover, unlike the above miRNAs, some miRNAs induce immunosuppressive microenvironments. For example, miR-146a facilitates M2-like macrophages marker genes expression and restricts M1-like macrophages marker gene expression [84].

Secondly, circular RNAs (circRNAs) are a class of ncRNAs that do not contain a 5′ end cap and a 3′ end poly tail [83]. It is widely involved in the regulation of tumor cell proliferation, differentiation, invasion, migration, and the formation of TME [85, 86]. For example, circCdyl promotes M1 polarization by inhibiting interferon regulatory factor 4 entry into the nucleus [87]. In addition, circPPM1F promotes lipopolysaccharide (LPS)-induced M1-like macrophages activation by enhancing the NF-κB signaling pathway [88]. It is different from the above two circRNAs, overexpression of hsa_circ_0005567 inhibited M1 polarization and promoted M2 polarization via the miR-492/SOCS2 axis [89].

Finally, long noncoding RNAs (lncRNAs) are a new class of RNA that is longer than 200 nucleotides and does not have protein-coding capabilities [90]. Some lncRNAs are involved in tumorigenesis and progression by regulating the TME. LncRNA-cox-2 inhibits tumor growth and immune evasion of HCC cells by inhibiting M2 polarization and promoting M1 polarization in macrophages [91]. LncRNA-CASC2c inhibits M2 polarization and tumor growth by inhibiting the expression and secretion of coagulation factor X [92]. LncRNA-TUC339 is highly expressed in M2-like macrophages and less expressed in M1-like macrophages. It is involved in the polarization of M2-like macrophages [93]. The epigenetic mechanisms that control macrophage polarization are complex. Enzymes and ncRNAs which play an essential role in gene modification, are expected to become new tumor markers and potential targets, providing new directions for tumor diagnosis and targeted therapy.

## Analysis of TAMs at the single-cell level

As mentioned earlier, TAMs have a wide range of plasticity and heterogeneity. However, traditional sequencing methods often mix a group of cells together for sequencing, making it difficult to capture possible heterogeneity between cells. Individual cell mutations in tumor progression cannot be accurately tracked [94]. To a large extent, the single-cell RNA sequencing (scRNA-seq) technology can solve this problem.

### Introduction of scRNA-seq

ScRNA-seq is a single-cell transcriptome analysis technique. The workflow typically includes sample collection, cell dissociation, single-cell capture, reverse transcription, cDNA amplification, library preparation, and sequencing and analysis [95]. ScRNA-seq enables quantitative analysis of gene expression profiles of different types of cells at the single-cell level, enabling unprecedented detail to characterize cell diversity and heterogeneous phenotypic status [96, 97]. This technique overcomes the shortcomings of traditional sequencing technology that cannot detect cell-cell heterogeneity and is an effective tool for studying gene expression patterns.

### Research advance of scRNA-seq technology for TAMs

The use of scRNA-seq for TAMs research mainly focuses on the following aspects: Firstly, identification of different macrophage subsets; Secondly, construction of the TME maps; Thirdly, identification of potential prognostic markers; Fourthly, analysis of intercellular interactions in TME; Finally, Interpretation the mechanisms of TAMs in tumor treatment and drug resistance (Table 2).

**Table 2:** Application of scRNA-seq in tumor-associated macrophages (TAMs)

Research Field	Cancer	Findings	References
Identification of different macrophage subsets	Small cell lung cancer	This study identified a profibrotic, immunosuppressive monocytes/macrophage population that is particularly associated with a PLCG2 high small cell lung cancer subpopulation.	[98]
	Breast cancer	ScRNA-seq reveals two subsets of APOE^+^ macrophages: the TREM2^+^ macrophages and the FOLR2^+^ macrophages. FOLR2^+^ macrophages are tissue-resident cells.	[99]
	Renal tumor	The study found a novel, tumor-specific sub-population of macrophages and differentially expressed genes (i.e., C1QA-C, APOE, and TREM2).	[100]
Construction of the tumor microenvironment maps	Gallbladder cancer	M2-like macrophages, epithelial cells, and Treg were predominant in ErbB pathway mutation tumors.	[101]
	Breast cancer	Most of the non-cancer cells are immune cells, with three distinct clusters of T lymphocytes, B lymphocytes and macrophages. Macrophages have an M2 phenotype that expresses many M2 genes, such as CD163, MS4A6A, and TGFBI, as well as genes known to promote tumor progression and angiogenesis, such as PLAUR13 and IL-8, exhibit immunosuppressive signatures.	[102]
	Glioma	Microglia-derived TAMs dominate in newly diagnosed tumors. However, they are overtaken by monocytes-derived TAMs after tumor recurrence, particularly in hypoxic tumor settings.	[103]
Identification of potential prognostic markers	Glioma	Sex-specific gene expression in glioma-activated microglia (e.g., genes encoding MHCII complexes) may be associated with morbidity and outcomes in patients with gliomas.	[104]
	Breast cancer	FOLR2^+^ macrophages positively correlate with better prognosis.	[99]
	Renal tumor	TREM2/APOE/C1Q^+^ macrophages infiltration is a potential prognostic biomarker for clear cell renal carcinoma recurrence.	[100]
Analysis of intercellular interactions in TME	Gallbladder cancer	High levels of midkine, expressed by the ErbB pathway mutation tumor cells, interact with the receptor LRP1 of tumor-infiltrating macrophages and promote immunosuppressive macrophage differentiation. The crosstalk between CXCL10 secreted by macrophage and its receptor CXCR3 on Tregs was induced in gallbladder cancer with ErbB pathway mutations.	[101]
	Breast cancer	FOLR2^+^ macrophages interact with tumor-infiltrating CD8^+^ T cells.	[99]
	Metastatic ovarian cancer	Macrophages in stress-high samples exhibited significantly higher expression of immunosuppressive features (C1QA, C1QB, C1QC, APOE, and TREM2), wherein TREM2 is functionally associated with T cell exhaustion.	[105]
	Head and neck tumors	The main contributors of PD-L1 in the TME were dendritic cells and macrophages. PD-1-PD-L1 interactions appeared to be primarily mediated by macrophages. PD-L1^+^ macrophages spatially associate with CD8^+^ T cells in the head and neck squamous cell carcinoma microenvironment.	[106]
	Gastric cancer	This study uncovered macrophages may contribute to HLA-E-KLRC1/KLRC2 interaction with cytotoxic CD8^+^ T cells and natural killer cells.	[107]
	Colon cancer	In tumors, TAMs and dendritic cells, as the core of the predictive network harbor the most connections with other cell types.	[108]
Explore the mechanisms of drug intervention	Colon cancer	Two distinct TAMs subsets show differential sensitivity to CSF-1R blockade treatment with anti-CSF-1R preferentially depletes macrophage populations with an inflammatory signature but spare macrophage subset that expresses proangiogenic and tumorigenic genes.	[108]
	Pan-cancer	Anti-PD-1 therapy decreases the number of Arg-1^+^ TAMs while increasing Arg-1-TAMs. On a local scale, a new cell subpopulation rich in chemotaxis and interferon response genes is formed.	[109]
	Pancreatic cancer	Anti-CD47 treatment led to changes in TME with increased pro-inflammatory macrophages, while reduced anti-inflammatory macrophages.	[110]
	Metastatic lung cancer	Macrophages demonstrated an inversion in relative abundance during tumor response and resistance to treatment.	[111]
Explore the mechanisms of drug resistance	Metastatic ovarian cancer	Stress-associated cancer cells strongly associate with a shift toward immunocompromised states within macrophages and CD8^+^ T cells. This stress-associated state provides cancer cells with adaptation, promoting chemoresistance.	[105]
	Pan-cancer	Tumor vessel co-option is a resistance mechanism against anti-angiogenic therapy. Matrix-remodeling macrophages might assist invasive cancer cells to co-opt vessels. An M1-like macrophage subtype may keep vascular cells quiescent.	[112]
Explore the mechanisms of non-drug interventions	Pancreatic cancer	After radiofrequency ablation, the percentage of Mac_s5 lacking mature markers decreased significantly; The proportion of Mac_s1 with anti-inflammatory gene expression profiles was also significantly reduced, and the proportion of Mac_s2 and Mac_s3 cells with anti-tumor functions increased.	[113]

*Abbreviations*: *TAMs* Tumor-associated macrophages

ScRNA-seq technology is used to identify different macrophage subsets and construct TME maps. In the analysis of macrophages in various tumors using scRNA-seq, macrophages are found to rely on different activation stimuli to obtain heterogeneous phenotypes. There are significant changes in gene expression in macrophages in TME compared to normal tissue [114–116]. For example, in a study of single-cell sequencing and protein activity of monocytic macrophages in kidney cancer tissues and adjacent tissues, a population of CD11C^+^ /CD163^+^ macrophages was identified to be higher than normal tissues in tumor tissues [100]. Compared to other cell populations and non-tumor macrophages, TAMs have unique differentially expressed genes (C1QA-C, APOE, and TREM1) and differentially active proteins (LILRB5, APOE, and TREM2) [117, 118]. Furthermore, scRNA-seq technology is used to construct TME maps. Single-cell transcriptome analysis of tumor tissue allows the characterization of heterogeneous tumor cells, adjacent stromal cells, and immune cells [102]. For example, M2-like macrophages, epithelial cells, and Treg were predominant in ErbB pathway mutation tumors [101]. In addition, the composition of TME varies at different stages of the tumor. The study found that microglia-derived TAMs dominate in newly diagnosed tumors. However, they are overtaken by monocytes-derived TAMs after tumor recurrence [103]. A good understanding of the TME maps of different tumor subtypes helps develop effective treatment strategies.

ScRNA-seq technology is used to identify potential prognostic markers. Different subpopulations of TAMs have unique marker genes that are sometimes linked to a patient’s prognosis [119]. Studies have reported that sex-specific gene expression in glioma-activated microglia (e.g., genes encoding MHCII complexes) may be associated with morbidity and outcomes in patients with gliomas [104]. Reliable prognostic markers guide physicians to understand disease trends and make rational clinical decisions.

ScRNA-seq technology is used to analyze cell-cell interactions in TME. As the central node of the cell-cell interaction network, TAMs play a vital role in the signal communication of TME. When tumors progress, FOLR2^+^ TAMs acquire the ability to activate naïve CD8^+^ T cells. FOLR2^+^ TRMs prime naive CD8^+^ T cells into polyfunctional effectors [99]. Understanding cell-cell communication networks helps us to use appropriate strategies to reshape TME.

ScRNA-seq technology is used to interpret the mechanisms of TAMs in tumor treatment and drug resistance. TAMs are one of the effector cells in various tumor treatment options. One of the mechanisms of action of many drugs to treat tumors is to alter TME by influencing TAMs. For example, after anti-CD47 treatment, the proportion of macrophages decreased while the proportion of lymphocytes increased, significantly reducing tumor growth [110]. TAMs also play an essential role in non-drug antineoplastic therapy. Radiofrequency ablation (RFA) is an effective local therapy approach for treating solitary tumors [120]. RFA treatment reduced the proportion of immunosuppressive cells, including TAMs, while increasing the percentage of functional T cells in distant non-RFA tumors [113]. Furthermore, TAMs also act a huge role in tumor drug resistance. Tumor vessel co-option is a resistance mechanism against anti-angiogenic therapy. Studies have shown that Matrix-remodeling macrophages might assist invasive cancer cells to co-opt vessels [112]. Clarifying the mechanism of action and resistance of drugs help guide the rational use of drugs in clinical practice and maximize the benefits for patients.

## Interaction effect of TAMs and PD-1/PD-L1 inhibitors in TME

### Effects of TAMs on PD-1/PD-L1 expression and TME

**Fig. 2:**
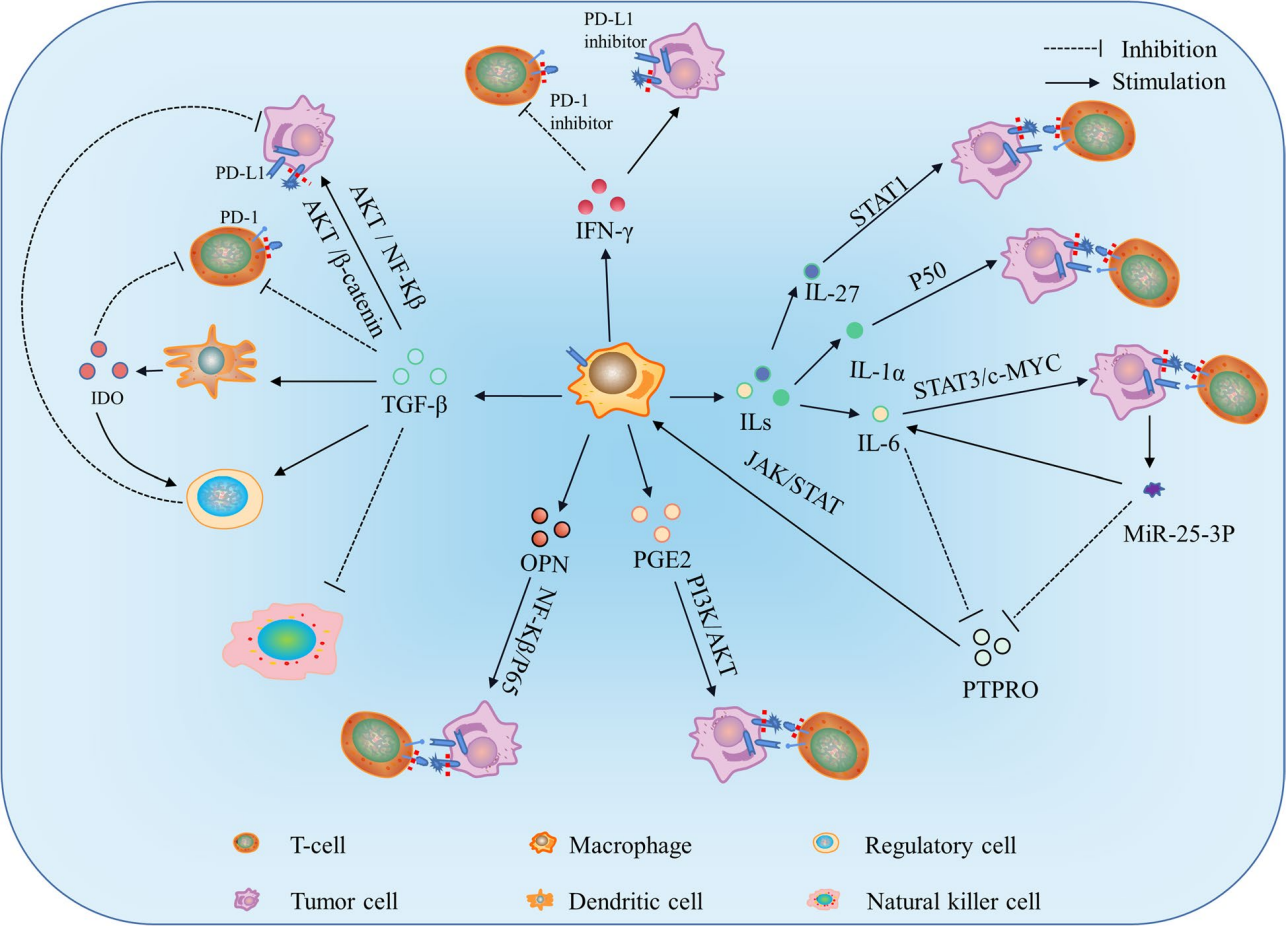
The release of multiple cytokines through TAMs affects PD-L1 expression. TGF-β upregulates tumor cell and PD-L1 expression of TAMs through the AKT/NF-kB or AKT/β-catenin pathway. IFN-γ upregulates PD-L1 expression by activating the transcription factor STAT1. PGE2 upregulates tumor cell PD-L1 expression by activating the PI3K/Akt/mTOR pathway. ILs upregulate PD-L1 expression in tumor cells through different pathways

As mentioned earlier, macrophages affect PD-L1 expression through various signaling pathways mediated by multiple cytokines. For example, TGF-β upregulates tumor cell and TAMs PD-L1 expression through the AKT/NF-kB or AKT/β-catenin (β-catenin plays a critical role in polarizing macrophages to TAMs, resulting in epithelial-mesenchymal transition and tumor progression) pathway after binding to its receptor [121, 122]. IFN-γ, a factor that promotes the transcription of new PD-L1 mRNAs by activating the transcription factor STAT1, facilitates PD-L1 transcription and translation rather than shifting PD-L1 stored intracellularly to the cell surface [123]. Under the action of pro-inflammatory factors such as LPS, IL-1β, and TNFα, TAMs synthesize large amounts of PGE2 [124]. PGE2 inhibits T cell activation and function by increasing PD-L1 expression. As a downstream of cyclooxygenase 2 (COX-2), PGE2 levels in TAMs are regulated by the expression of COX-2 and microsomal PGE2 synthase 1 [125]. In ovarian cancer cells, PGE2 upregulates the PD-L1 expression of tumor cells by activating the PI3K/ Akt/ mTOR pathway [126]. Osteopontin (OPN) is expressed in both tumor cells and TAMs. OPN-expressing macrophages upregulate PD-L1 expression via regulating the NF-kB/p65 pathway and aggravate tumor progression [127]. Interleukins of TAMs, such as IL-1a, IL-10, IL-27, IL-6, etc., also significantly influence the expression of PD-L1. Among them, IL-1a and IL-27 induce the transcription of new PD-L1 mRNA, thereby increasing the expression of PD-L1. The combined application of IL-1a/ IL-10 and IFN-γ enhances the expression of PL-L1, indicating synergy between different signaling pathways. There is no combined enhancement effect for IL-27 [128]. IL-1a signaling drives PD-L1 protein expression through p65, while IL-27 signaling drives PD-L1 protein expression through STAT1. IL-6 promotes PD-L1 expression in macrophages by regulating protein tyrosine phosphatase, receptor type O (PTPRO), either directly or indirectly through an IFN- γ -dependent mechanism. PTPRO is a negative regulator of the JAK2/STAT3 signaling pathway that induces immunosuppression. MiR-25-3p reduces transcription and protein expression by targeting the 3’UTR of PTPRO in macrophages. IL-6 upregulates miR-253p in tumor cells by STAT3/c-MYC signaling [129]. Furthermore, previous studies have shown that miR-25-3p secreted by tumor cells promotes IL-6 secretion in TAMs through exosomes [129] (Fig. 2).

### Effect of PD-1/PD-L1 on macrophages

Plenty of stimuli upregulate PD-1 expression. The upregulated PD-1 inhibits the Janus N-terminal-linked kinase signaling pathway and PI3K/Akt pathway by re-recruiting SHP-2. And then, PD-1 affects the function of macrophages and downregulates the expression of co-stimulatory molecules, such as CD86, MHC I, and II proteins [130]. Studies have reported that PD-1^+^ TAMs have a reduced phagocytic capacity compared to PD-1^-^ TAMs [131]. Moreover, the PD-L1 exosomes secreted by tumor cells have a positive feedback effect on the expression of PD-L1 in macrophages, which leads to M2 polarization of TAMs [132]. In addition, PD-1 has been shown to be associated with the apoptosis of macrophages. The expression of PD-1 in macrophages after hydrogen peroxide treatment is increased. Moreover, PD-1 negatively regulates the activation of the survival-promoting AKT pathway in macrophages through the PD-1-SHP-2 signaling axis, ultimately leading to increased macrophage apoptosis [133].

### Effect of anti-PD-1/PD-L1 therapy on macrophages

Studies have shown that anti-PD-1/PD-L1 therapy promotes macrophage maturation. After anti-PD-L1 treatment, the number of cell subsets lacking classic macrophage maturity markers such as Mertk in tumors decreases, while the number of Mertk-expressing cell subsets increases significantly. This increase is reflected in subpopulations and the overall number of macrophages [134, 135]. Furthermore, anti-PD-L1 therapy activates macrophages by upregulating the expression of the co-stimulatory molecules CD86 and MHC II. In addition, anti-PD-L1 treatment reduces the level of M2-like macrophages markers, such as Arg-1, on TAMs by promoting the production of IFN-γ in CD8 T cells. Meanwhile, anti-PD-L1 therapy increases the levels of M1-like macrophages markers such as iNOS, MHC II, and CD40 and promotes polarization of macrophages towards the pro-inflammatory phenotype. In addition, anti-PD-L1 therapy enhances the phagocytic ability of macrophages and the ability of macrophages to promote T cell activation and proliferation, increasing tumor clearance [136]. Meanwhile, it inhibits the polarization of macrophages to anti-inflammatory and immunosuppressive macrophages that promote tumor growth. The effect of this complex polarization is manifested both in changes of the function and molecular markers and in changes of genome-wide expression levels. Changes in genome-wide expression levels are mainly reflected in increased gene and protein expression of antigen presentation mechanisms. These include a variety of gene sets consisting of MHC molecules and phagocytosis-related Fcγ receptors, downstream IFN-γ, pro-inflammatory signaling, chemokine expression, TLR/NF-kB, and autophagy pathway upregulation [136].

## Effect of PD-1/PD-L1 inhibitor therapy combining with targeted agents/chemotherapy agents in solid cancers

**Fig. 3:**
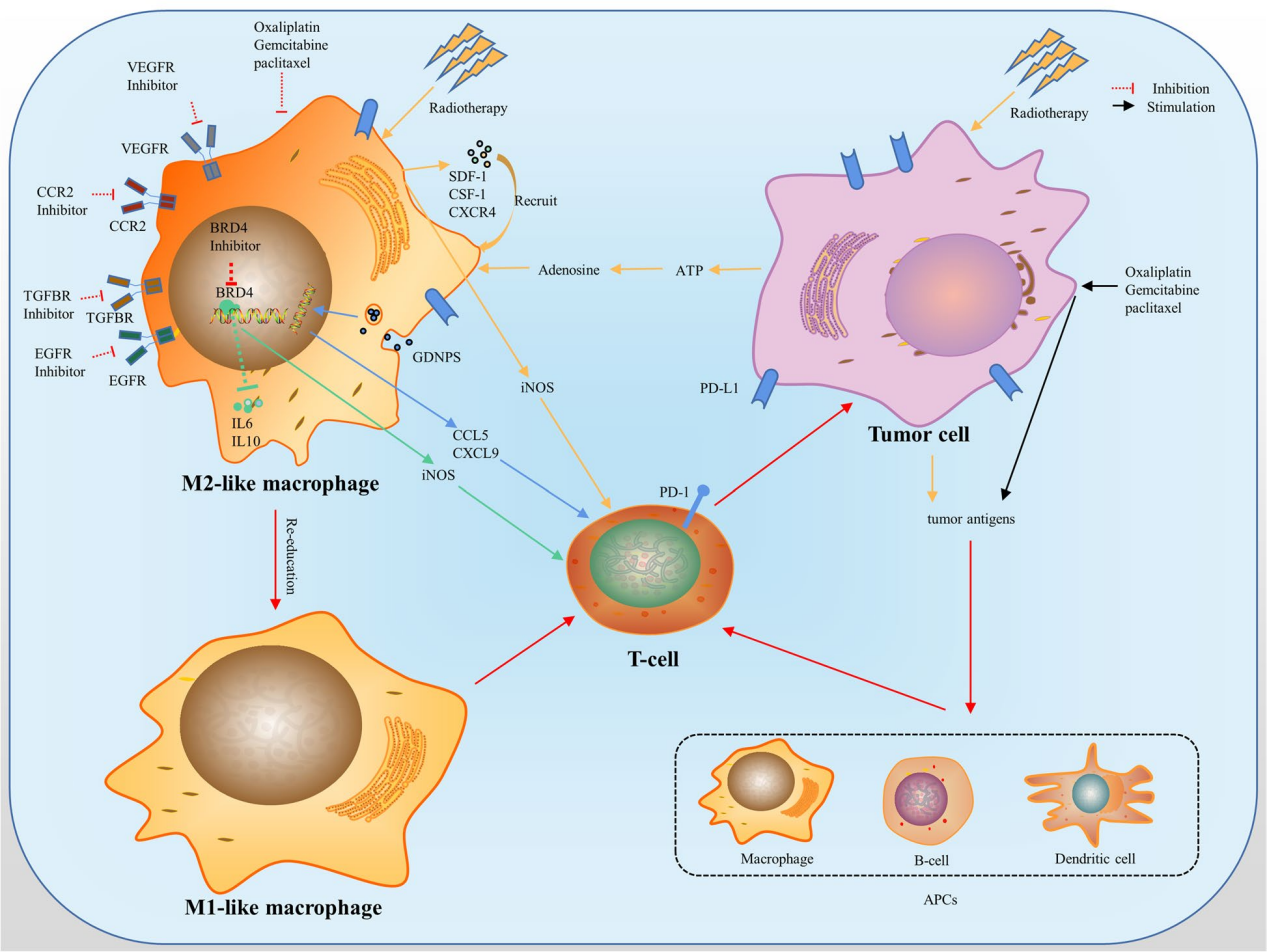
Combination therapy reverses M2-like macrophages into M1-like macrophages and activates T cells, promoting antitumor effects. Radiation therapy and chemotherapy induce tumor cell death and the release of tumor-associated antigens. Tumor-associated antigens are administered to T cells and activate T cells through antigen-presenting cells. Macrophages are re-recruited by tumor-associated antigens and radiation-induced cytokines. M1 polarization induced by chemotherapy drugs, radiation therapy, and targeted agents enhances immunotherapy sensitivity

As mentioned earlier, immune checkpoints induce immunosuppression. In addition to abnormal angiogenesis, immunosuppressive immune cells or cytokines, cancer-associated fat cells, and overactive cancer-associated fibroblasts modulate cancer immunity and promote immune tolerance [137–139]. On the one hand, studies have shown that removing the pre-existing immunosuppressive environment of TME enhances the efficacy of anti-PD-1/PD-L1 and helps to overcome primary drug resistance in cancer patients [140, 141] (Fig. 3). On the other hand, enhancing positive factors also improve the anti-PD-1 effect of PD-L1 therapy, such as immunogenic cancer cell death, immune support cytokines, and specialized antigen-presenting cells [142]. ICIs suppress the immune checkpoints and improve immunosuppression, while sometimes, a single ICI does not effectively activate the immune response. Traditional treatment options such as small molecule targeted drugs, chemotherapy, and radiation therapy improve immunosuppressive TME. Therefore, combining PD-1/PD-L1 inhibitors with these conventional therapies may improve the sensitivity to activate the antitumor immune response and the response rate of patients.

### Combining with targeted therapy

Targeted agents are mainly divided into antiangiogenic agents (bevacizumab, ramucirumab, or aflibercept) or anti-epidermal growth factor receptor (anti-EGFR) agents (cetuximab or panitumumab) according to different targets and other agents [143].

### Antiangiogenic agents

Angiogenesis abnormalities and immunosuppression in TME are two significant barriers to effective cancer immunotherapy [144, 145]. VEGF promotes the growth and survival of vascular endothelial cells. However, excessive angiogenesis may affect the maturation process of neovascularization and promote the formation of immunosuppressive TME [137, 138, 146]. In addition, cancer cells and stromal cells are reported to produce VEGF. VEGF upregulates the expression levels of PD-1 and other inhibitory checkpoints involved in CD8^+^ T cell failure and leads to non-response to anti-PD-1 therapy [147, 148]. Anti-VEGF receptor (anti-VEGFR) therapy relieves intraneoplastic hypoxia and immunosuppression by modulating abnormal tumor vessels. These above suggests that immunotherapy in combination with antiangiogenic agents may improve therapeutic outcomes [6, 149]. Lenvatinib is a multi-receptor tyrosine kinase inhibitor that suppresses its immunomodulatory function by targeting VEGFR with fibroblast growth factor signaling. Meanwhile, lenvatinib reduces the number of lowered M2-like macrophages in TAMs and increases the M1/M2 ratio. And lenvatinib increases the percentage of activated CD8^+^ T cells secreting IFN-γ and granzyme B (granzyme B is described as a critical soluble medium for cytotoxicity). In addition, lenvatinib upregulates plasmacytoid dendritic cell, the number of nuclear cells, especially CD8^+^ T cells, and their cytotoxic activity. Antitumor activity of lenvatinib plus anti-PD-1 combination therapy depends on lenvatinib’s activation of the IFN-γ signaling pathway [150]. In addition, lenvatinib alone and in combination therapy reduces the number of allogeneic tumor blood vessels, which may be another mechanism of lenvatinib combined with anti-PD-1 antitumor activity [150]. Apatinib is a small molecule targeted agent against VEGFR-2 that exhibits a dose- and time-dependent pattern for abnormal angiogenesis conditions. Low doses of apatinib plus anti-PD-L1 create a better immune support environment with more CD8^+^ T cells and fewer TAMs. Meanwhile, a more favorable pro-inflammatory microenvironment for immunotherapy appears 2 weeks after treatment [151].

### Anti-EGFR agents

EGFR is a transmembrane tyrosine kinase receptor involved in tumor cell proliferation, invasion, and metastatic angiogenesis [152]. Tyrosine kinase inhibitors (EGFR-TKIs) inhibit EGFR and alter the tumor immune microenvironment [153]. Cetuximab is a chimeric IgG1 monoclonal antibody that inhibits the EGFR intracellular signaling pathway by binding to the extracellular domain of EGFR [154]. Cetuximab binds to receptors on NK cells, causing NK cell activation and inducing their lytic activity against tumor cells. Tumor antigens are released after the lysis of tumor cells, which are then presented to CD8^+^ T cells via DC. Therefore, the effect of cetuximab is to increase the invasion of cytotoxic CD8^+^ T cells into tumors, enhancing the antitumor effect. However, it also induces upregulation of PD-L1 expression on tumor cells through negative feedback effects [155, 156]. It can be inferred from these mechanisms that the combination of anti-PD-1/PD-L1 and cetuximab may work through complementary mechanisms of action. The reason is that cetuximab is able to activate the immune system for avelumab therapy by recruiting CD8^+^ T cells into TME. And PD-1/PD-L1 inhibitors block the PD-1/PD-L1 signaling pathway.

### Anti-TGF-β agents

Dysregulation of TGF-β signal transduction pathways impairs multiple processes of the anticancer immune response, including antigen presentation, T cell infiltration, and tumor-killing activity. When anti-PD-1/PD-L1 alone does not work well in mouse colorectal tumor models, TGF-β blockers enhance the therapeutic efficacy of anti-PD-1/PD-L1. Blocking TGF-β1 and TGF-α2 significantly increased the Th1 immune response, upregulated IFN-γ production, and increased T-bet expression, a key transcription factor determining Th1 cell differentiation in tumor-infiltrating CD8^+^ T cells. An increasing number of Th1 cells promotes the polarization of TAMs towards M1-like macrophages and enhances the antitumor effect [157].

### Anti-transcription factors

Bromodomain containing 4 (BRD4), a member of the bromodomain and extraterminal protein family, interacts with the acetylated lysine residues of histone tails on chromatins. Oncogenic transcription factors (such as c-MYC) are amplified by recruiting transcription mechanisms or indirectly by binding to enhancers, contributing to cancer cell proliferation [158]. AZD5153 is an inhibitor of BRD4, which depolarizes M2-like macrophages. MAF is a critical TF in regulating macrophage phenotypes associated with ovarian cancer. AZD5153 significantly reduces the binding of BRD4 to MAF-TF in mouse high-grade serous ovarian cancer. By inhibiting the expression of M2-like macrophages-related genes, the proportion of M2-like macrophages is reduced without reducing the total number of TAMs, for that TAMs are polarized to M1-like macrophages [159].

### Nanotherapy

Cargo-free polymer nanoparticles (NPs) have a highly negative surface charge. When innate immune cells internalize NPs through scavenger receptors, including MARCO, they have an impact on the function of themselves. NP increases the production of TNF-α in tumor-bearing mice and reduces the expression of monocyte chemotaxis protein 1. The application of NP drugs reduces the aggregation of myeloid-derived suppressor cells at the tumor and metastatic sites. The combination of anti-PD-1/PD-L1 agents enhances this effect and promotes the efficacy of anti-PD-1/PD-L1 therapy [160]. Ginseng-derived nanoparticles (GDNPs) are isolated from Panax ginseng C.A. Mey. The expression of Ccl5 and Cxcl9 transcripts in M2-like macrophages increased significantly after GDNPs treatment. This promotes CCL5 and CXCL9 secretion, recruiting T lymphocytes to enhance tumor suppression. In addition, combination therapy with PD-1 monoclonal antibodies and GDNP reduced the M2/M1 ratio in tumors [161].

### Anti-ILT4

Immunoglobulin-like transcript-4 (ILT4) is an inhibitory receptor of the immunoglobulin superfamily. ILT4 is mainly expressed in myeloid cells, including DCs, granulocytes, monocytes, macrophages, and platelets [162]. EGFR activation induces ILT4 in non-small cell lung cancer (NSCLC) cells. ILT4 migrates TAMs to TME by promoting the secretion of CCLs (chemokine (C-C motif) ligands), such as CCL2 and CCL5. In addition, ILT4 induce M2 polarization, upregulating its markers including CD163, CD206, IL-10, and Arg-1, and downregulating M1-like markers in TAMs, including CD80, CD86, IL-12, and TNF-α. And ILT4 directly reduces the proliferation vitality and killing ability of T cells. ILT4 blockades inhibit the above functions of ILT4. The combination therapy of ILT4 blockades and PD-L1 inhibitors showed synergistic effects. The combination therapy not only significantly inhibits the migration of TAMs to TME and the expression of its surface markers but also increases the proliferation of T cells [163].

### Combining with chemotherapy

Chemotherapy mainly kills cancer cells and delays tumor growth by blocking the cell cycle, inhibiting DNA replication, interfering with cell metabolism, or inhibiting microtubule assembly. After cancer cell death, tumor antigens are presented by antigen-presenting cells, which leads to subsequent T cell recruitment, promotes the activation of the immune system, and thus promotes a highly effective antitumor immune response [8, 164, 165]. Induction of immunogenic cell death (ICD) is a critical way chemotherapy drugs work and can be induced by some anticancer drugs such as oxaliplatin. ICD requires cell surface CRT exposure, induction of EIF2α-dependent reticulum stress, HMGB1 and ATP release, and expression of type 1 IFNs (IFNα1 and IFNβ1) and chemokines (Cxcl9 and Cxcl0) [8, 165, 166]. The released molecules bind with its receptor to induce DC aggregation and enhance its antigen extraction ability, stimulating the adaptive antitumor immune response. In addition, chemotherapy drugs, such as gemcitabine and paclitaxel, increase the number of M1-like macrophages while reducing the number of M2-like macrophages and promote TAMs from M2-like macrophages to M1 repolarization [167]. 5-Fluorouracil (5-FU) selectively depletes bone marrow-derived suppressor cells in the body. 5-FU combined with oxaliplatin induces ICD in MSS colon cancer models and improves the efficacy of anti-PD-1, suggesting the possibility of using a combination of anti-PD-1 and chemotherapy to reverse immunotherapy resistance in MSS colon cancer [168–170]. Gemcitabine combined with anti-PD-1 antibody therapy increased CD8^+^ T cell infiltration compared with untreated and anti-PD-1 monotherapy. The same effect was observed in the treatment of pemetrexed. Chemotherapy exerts immunomodulatory effects by inducing immunogenic cell death, eliminating immunosuppressive cells, and enhancing effector cell function. Consequently, the TME modified by chemotherapy drugs favors anti-PD-1 antibody therapy.

## Radiotherapeutic effect on therapeutic sensitivity of PD-1/PD-L1 inhibitor through modulating TAMs in solid cancers

The mechanism of action of radiotherapy is similar to that of some chemotherapy drugs. Radiation therapy (RT) induces double-stranded DNA damage and leads to cell death through apoptosis, necrosis, autophagy, mitosis catastrophe, or replicating aging. Then, after death, tumor cells release tumor-associated antigens, triggering and stimulating immune responses. Radiotherapy provides a supportive local immune microenvironment for antitumor immunity and enhances systemic antitumor immunity, resulting in the regression of unirradiated distant tumors [171–174]. When combined with immunomodulatory drugs, irradiation may enhance changes in infiltrating immune cells [175]. This combination therapy of RT and PD-1/PD-L1 inhibitor improved the long-term survival in preclinical studies and mouse models of melanoma, colorectal cancer, breast cancer, and NSCLC [175–177], while also preventing tumor recurrence. It has also shown good promise in some clinical trials: 1. RT increases the effectiveness of PD-L1 inhibition, and 2. In combination with PD-L1 inhibitors, RT increases the patients’ survival [178, 179].

Macrophages, which show a high degree of plasticity under immune stimulation, are a critical direct effector cell in combination therapy [180, 181]. Radiotherapy upregulates chemo-attractant stromal cell-derived factor 1 (SDF-1) and CSF1as well as CXCR4 to enhance the infiltration of TAMs in tumors [182, 183]. Studies have shown that radiotherapy increases the phagocytosis of TAMs, which is consistent with a significant decrease in PD-1 expression of TAMs after low irradiation doses (PD-1 inhibits the phagocytosis of TAMs and changes in M1 polarization). Meanwhile, compared with unirradiated TAMs, irradiation promotes the ability of TAMs antigen presentation. This is the ability of CD86 expression in the irradiation group to increase significantly and polarize towards M1-like macrophages [131, 181]. After radiotherapy, the proportion of HLA-DR high expression in TAMs was increased considerably. And the low expression of HLA and human MHC was associated with poor clinical results. Meanwhile, RT promoted the secretion of cytokines such as IL-23 p19 and IL-12 p70, and the changes in cytokine profile had an essential impact on the polarization of TAMs [181, 184].

In addition, irradiation has conflicting effects on macrophage phenotypes. In some studies, low-dose irradiation (2 Gy) reduces the number of M2-like macrophages and induces repolarization of M2-like macrophages to M1-like macrophages by increasing the expression ofiNOS, enhancing antitumor effects [181, 185]. In some other studies, irradiation was reported to lead to an increase in CD68^+^CD163^+^ M2-like macrophages around the tumor in NSCLC patients. The mechanism might be the release of ATP caused by RT-induced cancer cell death, which in turn was decomposed into adenosine. The accumulation of extracellular adenosine leads to the polarization of TAMs to M2-like macrophages [186–188]. In addition, RT induces ROS production. ROS-induced oxidation in the latency-associated peptide further activates TGF-β. TGF-β directly promotes the M2 polarization of TAMs. And TGF-β also upregulates the expression of immunosuppressive genes in M2-like macrophages, such as genes encoding IL-17 receptors (IL-17RB), to promote the development of Th17 cells. TGF-β also increases the expression of the outer nucleotides CD73 and CD39 of Th17 cells by down-regulating zinc finger protein growth factor independent-1 and inducing STAT3 expression, respectively. The overall manifestation is an increase in the number of Th17 cells and the expression of genes in Th17 cells responsible for converting ATP to adenosine [189]. HIF-1α has been shown to cause radiation resistance in endothelial cells, causing angiogenesis and tumor progression by promoting the expression of VEGF-A [190, 191]. RT stabilizes HIF-1α in cancer cells and thus increases cell content directly. RT also indirectly stabilizes HIF-1α by increasing TAMs [190].

The effectiveness of radiation therapy depends on several aspects. Tumor type and histotype: preoperative radiotherapy induces upregulation of PD-L1 in patients with cervical gland/adenosquamous cell carcinoma and soft tissue sarcom. While more patients with NSCLC or rectal cancer have decreased PD-L1 expression [181, 192–194]; Different combination regimens: a retrospective analysis of patients with metastatic melanoma reported a response rate of 64% in patients treated with both stereotactic radiosurgery and anti-PD-1 antibodies, higher than the 44% response rate in patients treated sequentially [195]; Irradiation dose: RT also modulates the immune system and TME in a dose-dependent manner. In some studies, low-dose RT promotes antitumor immunity. For example, low-dose RT of rectal cancer tissue differentiates TAMs towards the pro-inflammatory M1-phenotype, and high-dose RT at doses of 12–18 GY has also been shown to weaken the effect of antitumor immunity [181, 196]. In general, RT induces both immune activation and immunosuppression. When the impact of RT on TME is to enhance antitumor immunity, it will undoubtedly promote the effect of immunotherapy. If RT induces immunosuppressive effects, such as upregulating PD-L1, the combination of anti-PD-1/PD-L1 antibodies alleviates this immunosuppression. In addition, simultaneous administration helps increase the response rate and the effect of aspirin. These above suggests that the combination of radiotherapy and immunotherapy is promisingly more valuable [195, 197, 198].

## Effect of listeria vaccine on cancer therapeutic efficiency of PD-1/PD-L1 inhibitors by regulating macrophages

As mentioned earlier, the low antigenicity of cancer cells and poor penetration and accumulation of immune cells in the TME are important reasons for the poor response to immune checkpoint therapy [199]. Cancer vaccines enhance immunogenicity, activate the patient’s immune system, and improve the effect of immune checkpoint treatment [200]. Cancer vaccines are another representative strategy for cancer immunotherapy, mainly divided into two main categories: preventive and therapeutic. Prophylactic vaccines induce immune memory by vaccinating healthy people to prevent the occurrence of specific cancers. The role of therapeutic vaccines is to strengthen or activate a patient’s immune system to treat cancer patients [201]. The development of cancer vaccines is based on the clinical phenomenon that patients with some infectious diseases are less likely to develop cancer than the general population [202]. For example, some specific antibodies produced by people with mumps reduce the incidence of ovarian cancer, and BCG vaccines used to prevent tuberculosis are now doing well in bladder cancer treatment [202, 203]. The mechanism of cancer vaccine treatment of tumors is to artificially stimulate and induce tumor antigen-specific T cells by using foreign antigens. As a result, TME is optimized, which induces cancer-specific immune responses [204].

However, immunosuppressive TME makes cancer vaccines less effective alone. The advantages and disadvantages of cancer vaccines and anti-PD-1/PD-L1 immunotherapy complement each other to a certain extent. Studies have shown that a Listeria-based HCC vaccine (Lmdd-MPFG), an oncology vaccine based on *Listeria monocytogenes*, elicits a strong anti-HBV-associated HCC immune response [205]. Its combination therapy with anti-PD-1/PD-L1 therapy has exerted a huge synergistic effect. There have been some changes in TME after vaccination. At the cellular level: Lmdd-MPFG vaccine is a potent macrophage polarizer. Lmdd-MPFG vaccine activates the NF-kB pathway via the TLR2 and MyD88 pathways and upregulates autophagic proteins, such as Atg16L1, Beclin1, LC3-II, p62, to enhance the autophagy process in M0 or M2-like macrophages. As a result, TAMs were repolarized from M2-like to M1-like macrophages. And CD8^+^ T cells with antitumor effects and DCs infiltration were significantly increased, while Treg cells (CD4^+^, CD25^+^, FoxP3^+^) were significantly reduced. The result is T cells resensitization to immune checkpoint blocking therapy [205]. At the cytokine level: an increase in M1-like macrophages were accompanied by an increase in gene expression of M1-like macrophages-related cytokines and chemokines, such as IFN-γ, iNOS, IL-23, CCl2, IL-1b, TNF-α. Meanwhile, the level of M2-like macrophages markers, such as IL-10, Arg-1, CD206, Fizz, TGF-β, Mgl-2, PDCD1LG2, and Ym-1 are reduced [205]. However, Lmdd-MPFG induces upregulation of PD-L1 expression levels in tumor tissue, while combined PD-1 antibodies enhance T cell responses by eliminating overexpression of PD-L1 in tumor tissues that may be induced by vaccines.

## Roles of TAMs in immune therapy of PD-1/PD-L1 inhibitors for different solid cancers

**Fig. 4:**
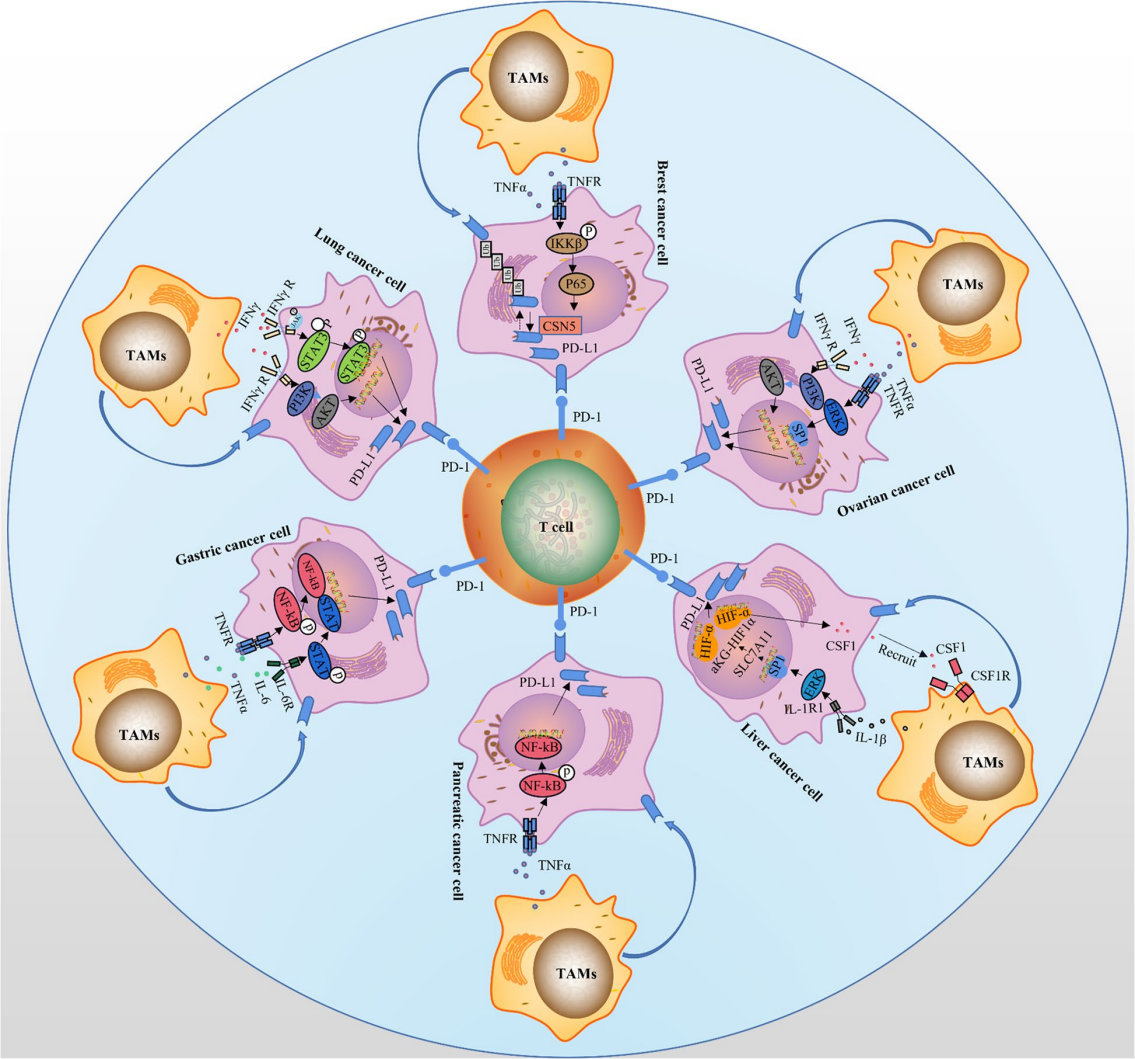
The effect of TAMs in different solid cancers. In pancreatic cancer, TAMs promote PD-L1 expression in cancer cells through TNF-α/NF-kB pathway. In liver cancer, TAMs promote PD-L1 expression in cancer cells through the IL-1β/ERK pathway. In gastric cancer, TAMs promote PD-L1 expression in cancer cells through IL-1/STAT and TNF-α/NF-kB pathways. In lung cancer, TAMs promote PD-L1 expression in cancer cells through IFN-γ/PI3K/AKT and IFN-γ/STAT3 pathways. In breast cancer, TAMs promote PD-L1 expression in cancer cells through TNF-α/IKK pathway. In ovarian cancer, TAMs promote PD-L1 expression in cancer cells through IFN-γ/PI3K/AKT and IFN-γ/ERK1 pathways

In different solid cancer types, TAMs often affect the expression of PD-1/PD-L1 through different pathways, which in turn affects the efficacy of PD-1/PD-L1 inhibitors (Fig. 4).

### Pancreatic cancer

In pancreatic cancer, PD-L1 expression is correlated with CD163^+^ TAMs. TNF-α significantly increases the expression of PD-L1 mRNA, compared with other cytokines secreted by TAMs, such as IL-1a, IL-1b, IL-4, IL-6, IL-7, etc. The specific mechanism is that TNF-α increases the phosphorylation level of NF-kB. And real-time PCR confirms a clear positive correlation between PD-L1 protein expression and TNF-α mRNA level in PDAC tissues [206].

### Liver cancer

In liver cancer, IL-1b, secreted by TAMs, upregulates the expression of SLC7A11 through the IL-1R1 pathway. SLC7A11 reduces the level of α-ketoglutarate by transferring intracellular glutamic acid to the extracellular, thereby reducing the degradation of HIF-1α and increasing its content in the cell. At the same time, SLC7A11 activates the AKT pathway and induces HIF-1α expression under low oxygen conditions [207]. HIF-1α, as a critical positive regulator of PD-L1 expression, binds with the HIF-1α binding site on the PD-L1 promoter, thereby upregulating PD-L1. In addition, SLC7A11 overexpression promotes the infiltration of TAMs in tumors through the CSF1 receptor (CSF-1R) axis [207, 208].

### Gastric cancer

When co-cultured with gastric cancer cells, the expression of TNF-α and IL-6 in TAMs increased significantly. Cytokines of IL-1 and IL-1b do not have this trend. TNF-α and IL-6 induced PD-L1 expression through NF-kB and STAT3 signaling pathways [209].

### Lung cancer

Studies have shown that in lung cancer, TAMs produce IFN-γ, IL 6, TNF-α, and IL 10 to induce A549 to express PD-L1. And IFN-γ induces tumor cells to express PD-L1 more effectively than other cytokines. It is the primary molecule induced by PD-L1 by TAMs in lung cancer. The mechanism that TAMs increase the secretion of IFN-γ is achieved by upregulating the JAK/STAT3 and PI3K/AKT signaling pathways [61].

### Breast cancer

Compared with cytokines such as IL-6, IL-8, IL-1, and TNF-α secreted by macrophages, upregulates the expression of PD-L1 protein through non-transcriptional regulatory mechanisms (such as post-translational regulation) without increasing mRNA expression. The specific mechanism is that TNF-α binds to its ligand to activate IKKb-kinase, which induces a nuclear shift downstream p65. p65 interacts directly with COPS5, the promoter encoding CSN5, transcriptionally upregulating its expression. CSN5 is a subunit with deubiquitinase activity in the COP9 signaler. It interacts with PD-L1 and deubiquitinates it to stabilize it [210].

### Ovarian cancer

In ovarian cancer, TAMs-derived IFN-γ, TNF-α, IL-10, and IL6 increase PD-L1 expression, and the density of membrane PD-L1 is positively correlated with high TAMs infiltration. The expression of PD-L1 was induced through IFN-γ via the PI3K pathway. And TNF-α induced the expression of PD-L1 through the ERK1/2 pathway [211].

Overall, existing evidence suggests that PD-L1 may be differentially regulated with respect to specific signaling pathways and transcription factors in different cell types. This provides guidance for the precise treatment of various cancers.

## The clinical application value of TAMs in solid cancer therapy

In ICIs-mediated therapy, TAMs play a very important bifacial role, antitumor and pro-tumor. Because of this, targeting macrophage synergistic ICIs is a promising combination protocol. At present, macrophage-centred therapeutic strategies mainly divide into four aspects.

### Reduces the recruitment of macrophages

CSF1and CCL2 play a crucial role in monocyte recruitment and TAMs generation [212]. For example, blocking CCL2/CCR2 restricts monocytes from entering the tumor [213, 214]. Inhibition of CSF1 reduces TAMs invasion as well as tumor proliferation and migration [215]. In addition to the CCR2-CCL2 signaling axis, CXCR4-CXCL12 (also known as stromal cell-derived factor-1, SDF-1) interaction is another signaling axis involved in the recruitment of monocytes/macrophages and implicated in the promotion of tumor invasiveness/regrowth [183]. Treatment with a CXCR4 inhibitor (AMD3100) inhibits its effect.

### Depletion of existing macrophages in the TME

CSF-1R is a tyrosine kinase receptor expressed on mononuclear phagocytes [216]. After binding to CSF1, CSF-1R promotes the proliferation, function, and survival of macrophages. CSF-1R antibodies deplete TAMs by blocking the function of CSF-1R [216]. Certain drugs, such as Bisphosphonate, also induce apoptosis after being swallowed up by TAMs [217]. Mannose receptor (CD206) is overexpressed on M2-like macrophages, which is also one of the most commonly targeted receptors of macrophages [218]. Chimeric antigen receptor T-cell (CAR-T) immunotherapy specifically kills target cells. Construction of CAR-T specific against immunosuppressive subsets in TAMs reduces the number of TAMs in TME [219, 220].

### Repolarization of existing macrophages in the TME

Multiple means polarize the TAMs to the M1 type. For example, immunomodulators, especially monoclonal antibodies, are widely used as monotherapy and as adjuvants conditioning TME for combinatorial treatments. An anti-MARCO (A pattern recognition scavenger receptor) induces anti-tumor activity in multiple tumor models by reprogramming the TAMs population into a pro-inflammatory phenotype and increasing tumor immunogenicity [221]. LILRB4 is a LILRB family receptor that is widely expressed on immune cells and enriched on TAMs [222]. After treatment with anti-LILRB4 antibodies, TAMs transitions to a less inhibitory phenotype [223]. In addition, as mentioned earlier, radiotherapy, targeted therapy and a variety of chemotherapy drugs have a similar effect [73, 150, 151, 167].

### Macrophage cell therapy

Engineered receptors are used to arm monocytes to treat tumors. Based on the ability of macrophages to infiltrate tumors and their unique ability in TME, the method of genetically engineering macrophages with CARs to enhance their ability to kill tumors has great potential [224, 225]. While killing tumor cells, CAR macrophages (CAR-Ms) also inhibit the M2 polarization of TAMs and promotes M1 transformation. In addition, the expression of the CAR structure reverses the M2 polarized macrophages to the M1-like macrophages [225].

Monocytes are used as vehicles to deliver cytokines or nanoparticles to TME. Tie2-expressing monocytes have tumor-homing ability. It is used as a vehicle to deliver the anti-tumor cytokine IFNα to TME, which inhibits tumor angiogenesis and activates innate and adaptive immune cells [226].

## Conclusions

As previously mentioned, TAMs are essential in treating solid tumors through multiple mechanisms. TAMs can regulate the expression of PD-L1 molecules in tumor cells through various pathways. Meanwhile, TAMs are significant targets of PD-1 / PD-L1 inhibitors and critical cells in mediating the role of traditional therapeutic options such as radiotherapy, chemotherapy, and targeted therapy. The above functions of TAMs are a critical basis for combining immunotherapy with conventional treatment regimens. However, the understanding of TAMs needs to be further improved. The urgent problems to be solved include 1. What are the key factors driving the phenotypic changes of TAMs in TME? 2. How to distinguish TAMs into subgroups with different functions and identify a subset of the required functions? Many studies have shown that combination therapy based on PD-1 / PD-L1 inhibitors with other traditional treatment regimens can synergistically benefit tumor patients. However, combination treatment regimens not only increase patient medical costs but also reduce the low toxicity of patients. Optimizing the combination treatment regimen, including drug, dose, timing, and order is a significant difficulty in developing combination treatment.

In conclusion, further studies on the classification and function of TAMs will help to improve the responsiveness of cancer patients to immune checkpoint therapy. Moreover, based on the immunosuppressive effects of TAMs, the development of drugs targeting TAMs to reduce the recruitment to TME and the clearance and repolarization of existing TAMs in TME are also hot research fields. Developing a good combination application program will also greatly promote the development of the tumor treatment field for the benefit of cancer patients.

## Data Availability

Not applicable.

## Abbreviations

Anti-EGFR: Anti-epidermal growth factor receptor
Anti-VEGFR: Anti-VEGF receptor
Arg-1: Arginase 1
ASPS: Alveolar soft part sarcoma
BCC: Basal cell carcinoma
BRD4: Bromodomain containing 4
BTC: Biliary tract cancer
CAR-T: Chimeric antigen receptor T-cell
CAR-Ms: CAR macrophages
CC: Cervical cancer
CRC: Colorectal cancer
CircRNAs: Circular RNAs
COX-2: Cyclooxygenase 2
CSCC: Cutaneous squamous cell carcinoma
CSF1: Colony stimulating factor 1
CSF-1R: CSF1 receptor
CTCF: CCCTC-binding factor
CTLA-4: Cytotoxic T lymphocyte-associated protein 4
DCs: Dendritic cells
DNMTs: DNA methyltransferases
EA: Esophageal adenocarcinoma
ESCC: Esophageal squamous cell carcinoma
ES-SCLC: Extensive-stage small cell lung cancer
GC: Gastric cancer
GEJC: Gastroesophageal junction cancer
GDNPs: Ginseng-derived nanoparticles
HATs: Histone acetyltransferase
HCC: Hepatocellular cancer
HDACs: Histone deacetylase
HL: Hodgkin lymphoma
HNSCC: Head and neck squamous cell cancer
ICD: Immunogenic cell death
ICIs: Immune checkpoint inhibitors
IDO: Indolamine 2,3-dioxygenase
ILT4: Immunoglobulin-like transcript-4
iNOS: Inducible nitric oxide synthase
Lmdd-MPFG: Listeria-based HCC vaccine
LncRNAs: Long noncoding RNA
LPS: Lipopolysaccharide
MCC: Merkel cell cancer
M-CSF: Macrophage colony-stimulating factor
MiRNAs: MicroRNAs
MM: Multiple myeloma
MPM: Malignant pleural mesothelioma
ncRNAs: Non-coding RNAs
NKs: Natural killer cells
NPC: Nasopharyngeal carcinoma
NPs: Nanoparticles
NSCLC: Non-small cell lung cancer
OPN: Osteopontin
PD-L2: Programmed death ligand 2
PMBCL: Primary mediastinal B-cell lymphoma
PTPRO: Protein tyrosine phosphatase, receptor type O
RCC: Penal cell cancer
RFA: Radiofrequency ablation
ROS: Reactive oxygen species
RT: Radiation therapy
SC: Skin cancer
SCLC: Small cell lung cancer
scRNA-seq: Single-cell RNA sequencing
TAMs: Tumor-associated macrophages
TGF-β: Transforming growth factor-β
TLR: Toll-like receptor
TME: The tumor microenvironment
TNBC: Triple-negative breast cancer
TNF-α: Tumor necrosis factor-α
UC: Urothelial cancer
VEGF: Vascular endothelial growth factor
